# First-in-human in-vivo depiction of paraganglioma metabolism by hyperpolarised ^13^C-magnetic resonance

**DOI:** 10.1259/bjrcr.20220089

**Published:** 2023-09-28

**Authors:** Rafat Chowdhury, Myuri Moorthy, Lorna Smith, Christoph A Mueller, Fiona Gong, Harriet J Rogers, Marianthi-Vasiliki Papoutsaki, Tom Syer, Giorgio Brembilla, Saurabh Singh, Adam Retter, Thomas Parry, Joey Clemente, Lucy Caselton, Hassan Jeraj, Max Bullock, Manju Mathew, Teng Teng Chung, Scott Akker, Paul Chapple, Grace A Salsbury, Alan Bainbridge, David Atkinson, David G Gadian, Umasuthan Srirangalingam, Shonit Punwani

**Affiliations:** 1 Centre for Medical Imaging, Division of Medicine, University College London, London, UK; 2 Department of Endocrinology, University College London Hospital, London, UK; 3 Department of Radiology, Faculty of Medicine, Medical Centre University of Freiburg, Freiburg im Breisgau, Germany; 4 German Cancer Consortium (DKTK), partner site Freiburg, German Cancer Research Center (DKFZ), Heidelberg, Germany; 5 Department of Endocrinology, St Bartholomew’s Hospital, London, UK; 6 William Harvey Research Institute, Barts and The London School of Medicine and Dentistry, Queen Mary University of London, London, UK; 7 Department of Medical Physics and Biomedical Engineering, University College London Hospitals NHS Foundation Trust, London, UK; 8 UCL Great Ormond Street Institute of Child Health, London, UK; 9 Department of Radiology, University College London Hospitals NHS Foundation Trust, London, UK

## Abstract

Phaeochromocytomas (PCC) and paragangliomas (PGL), cumulatively referred to as PPGLs, are neuroendocrine tumours arising from neural crest-derived cells in the sympathetic and parasympathetic nervous systems. Predicting future tumour behaviour and the likelihood of metastatic disease remains problematic as genotype–phenotype correlations are limited, the disease has variable penetrance and, to date, no reliable molecular, cellular or histological markers have emerged. Tumour metabolism quantification can be considered as a method to delineating tumour aggressiveness by utilising hyperpolarised ^13^ C-MR (HP-MR). The technique may provide an opportunity to non-invasively characterise disease behaviour. Here, we present the first instance of the analysis of PPGL metabolism via HP-MR in a single case.

## Summary

Phaeochromocytomas (PCC) and paragangliomas (PGL), cumulatively referred to as PPGLs, are neuroendocrine tumours arising from neural crest-derived cells in the sympathetic and parasympathetic nervous systems.^1^ Predicting future tumour behaviour and the likelihood of metastatic disease remains problematic as genotype–phenotype correlations are limited, the disease has variable penetrance and, to date, no reliable molecular, cellular or histological markers have emerged.^2^ Tumour metabolism quantification can be considered as a method to delineating tumour aggressiveness by utilising hyperpolarised 13C-MR. Evidence of lactate production, via pyruvate metabolism, and tumour grade in prostate cancer, with greater lactate seen in more aggressive, higher-grade tumours.^3^ The technique may provide an opportunity to non-invasively characterise disease behaviour.^4^ Here, we present the first instance of the analysis of PPGL metabolism via HP-MR in a single case.

## Case presentation

A male in his mid-60s, presented with bowel obstruction and was found to have a 12 cm heterogeneous right-sided adrenal mass in close apposition to the liver and inferior vena cava. He had a background of mild hypertension and originated from a high-altitude region in South America. Written informed consent to participate in a HP-MR study was obtained from the subject.

## Investigations

Elevated plasma metanephrines and a positive meta-iodobenzylguanidine (MIBG) isotope scan suggested a diagnosis of PCC without metastasis. Following appropriate adrenergic blockade with phenoxybenzamine and propranolol, an adrenalectomy was attempted in 2014 but could not be completed due to torrential bleeding from the liver capsule, allowing only a partial resection. Histology confirmed a partially resected 110 × 100 × 70 mm PCC, with no evidence of lymphovascular or capsular invasion, with Ki67 <1%. There were no signs of necrosis and succinate dehydrogenase B (SDHB) genetic testing returned negative. High cellularity was observed with spindle-shaped tumour cells, profound nuclear pleomorphism and nuclear hyperchromasia.

A second surgical attempt was again abandoned due to significant blood loss and haemodynamic instability despite adequate blockade. Genetic testing with a next generation sequencing panel of susceptibility genes was negative for: succinate dehydrogenase (SDHx) SDHD, SDHB, SDHC, SHAF2, RET, MAX, TMEM127, FH and VHL, failing to identify a causative mutation. There was no history of familial disease. The subject subsequently underwent three cycles of ^131^I-MIBG therapy in 2016 (total dose 32.37 GBq), for residual local disease, which was terminated following significant myelosuppression.

He was managed expectantly until surveillance in 2018 noted significant disease progression with spread to the peritoneum, lung, bone, para-aortic and mediastinal nodes. Imaging found that metastases were non-MIBG avid but were octreotide avid on ^68^Ga-Dotatate imaging. ^177^Lu-Dotatate and yttrium-90 (^90^Y) peptide receptor radionuclide therapy (PRRT) were not feasible therapy at the time and so he was started on palliative chemotherapy with temozolomide (TMZ) in 2019.

Due to the size of the PPGL, the subject was recruited and scanned as part of a hyperpolarised ^13^C-MR (HP-MR) feasibility study in December 2019, after his third cycle of TMZ therapy, as a first-in-human determination of the non-invasive assessment of PPGL metabolism using [1-^13^C] pyruvate.

A fluid path was prepared and hyperpolarised [1-^13^C] pyruvate extracted as previously described,^4^ with a polarisation level of 32.3% achieved after 3 h in a clinical hyperpolariser (SPINLab, GE Healthcare, Chicago, IL). The injection of the hyperpolarised [1-^13^C] pyruvate started 1 m 18 s after removal from the polariser (dissolution) with ^13^C-MR acquisition beginning immediately after completion (12 s).

**Figure 1.**
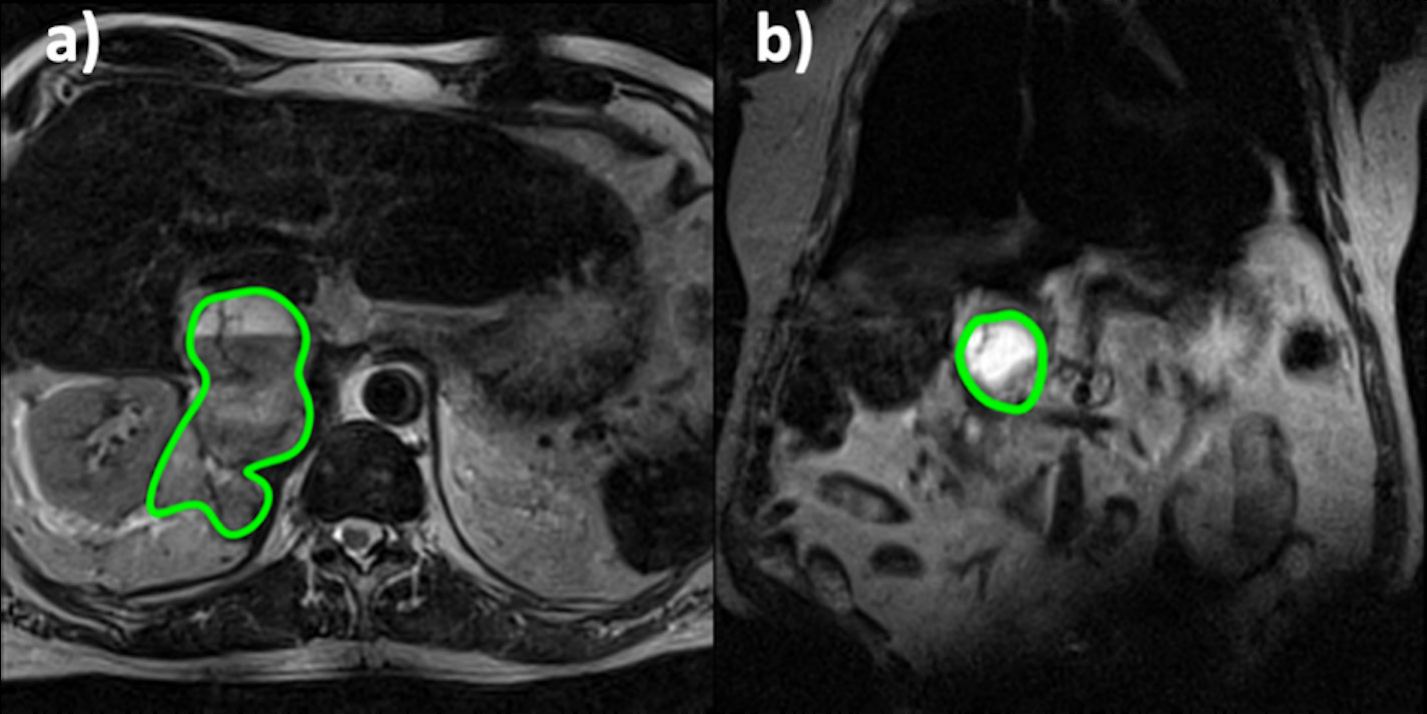
Axial (a) and coronal b) T_2_-weighted images were used to localise the tumour, outlined in green, prior to injection of hyperpolarised [1-^13^C] pyruvate. The brighter signal within the ROI can be attributed to a cystic tumour component.

The subject was positioned supine in a 3T positron emission tomography-MRI (PET-MRI; Siemens Biograph mMR) with a ^13^C-clamshell transmit and two seven-channel, dual-tune, ^1^H/^13^C receive phased array coils (RAPID Biomedical GmbH) with one each placed on the subject’s anterior and posterior. Prior to contrast injection the tumour position was localised using *T*_2_ weighted turbo spin echo (TSE) MR scans, performed in the axial and coronal planes (Figure 1). After this, a dual echo gradient sequence was employed to assess the magnetic field inhomogeneity across the field of view (see Supplementary Material 1 for all ^1^H-MR sequence parameters).

Supplementary Material 1.

Four consecutive multiecho-balanced steady-state free precession (ME-bSSFP) datasets were acquired, with free breathing, followed by a single non-localised spectroscopy acquisition, and this process was repeated every 13 s for a total acquisition time of 2 m 36 s (see Supplementary Material 1 for all ^13^C-MR sequence parameters). An iterative decomposition of a fat and water signals with least-squares estimation (IDEAL) model with bipolar echo correction, utilising an *a priori* field map was used to resolve the ME-bSSFP echo data, with the nonlocalised spectra used to identify the species of interest, thus affording maps of [1-^13^C] pyruvate and its downstream metabolites.^5^

Region of interest (ROI) analysis involved, firstly, identifying all voxels which overlaid the tumour on the metabolite maps. The mean signal (S_t_) of these voxels within the ROI was calculated at each time point. Then, the last five bSSFP measurements were isolated to derive a noise value (N); at this point, the signal from the hyperpolarised [1-^13^C] pyruvate (*t* = 150 s) was no longer present. The standard deviation of signals across all voxels within the ROI, across all five time points was calculated, affording N. The ratio of S/N was calculated (SNR) at each time point for all metabolites. SNRs were then summated, for every four measurements, with this being repeated for all metabolites.

## Results

**Figure 2.**
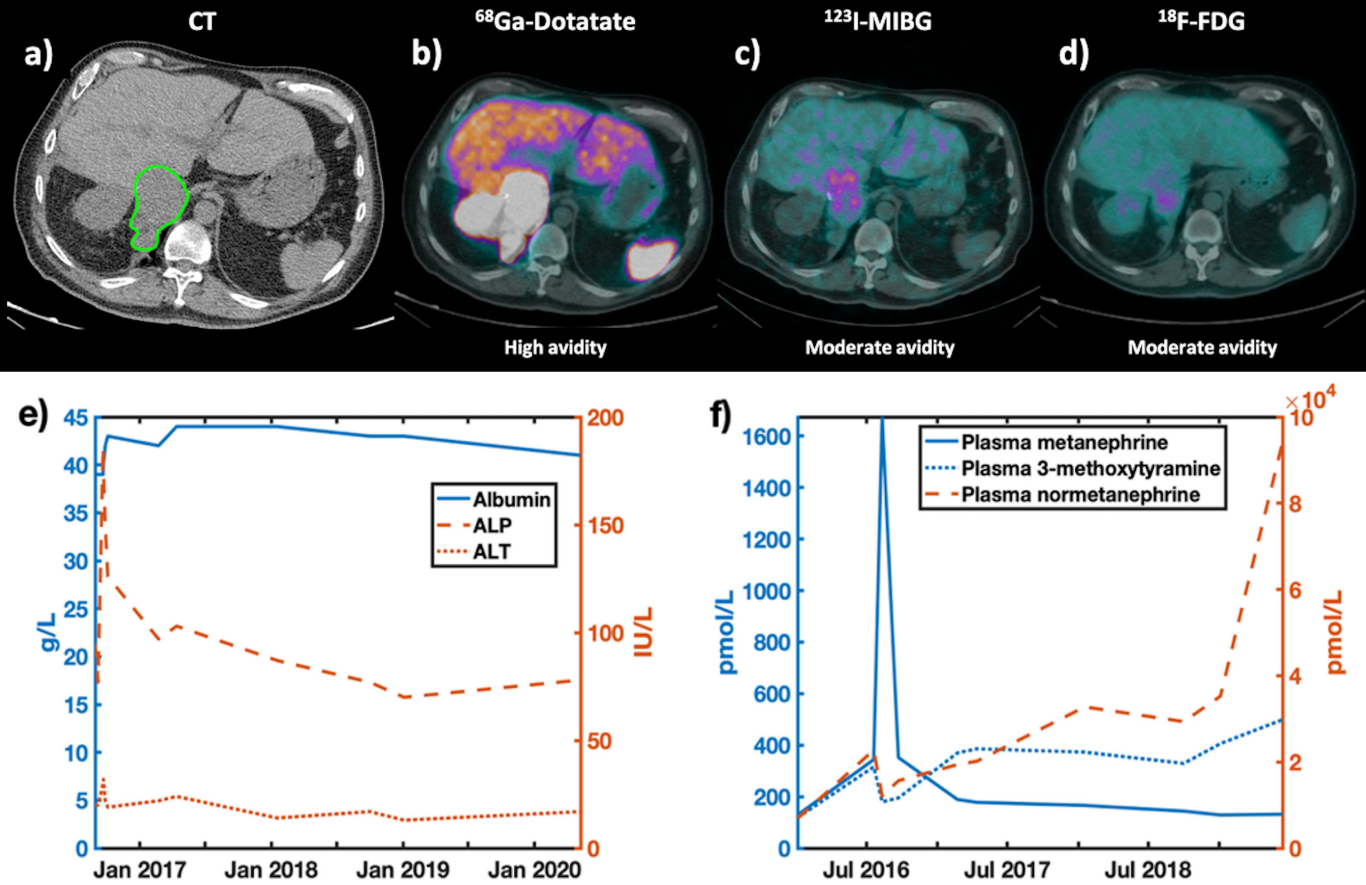
CT(a) and PET(b-d) imaging demonstrating the location of the pheochromocytoma. PET tracers included: b)^68^Gallium Dotatate, c) Iobenguane (MIBG), d) ^18^F-fluorodeoxyglucose (^18^FDG-PET). A qualitative assessment of tracer avidity is provided below each PET image. e) Blood albumin, ALP and ALT levels, as well as f) results for plasma metanephrine levels, showing levels of metanephrine, 3-methoxytyramine and normetanephrine are shown. These factors were considered to track disease stage.

CT and PET scans for the subject are shown (Figure 2a-d), with avidity varying by modality and tracer. The subject appeared to be ^18^F-FDG avid (Figure 2d), with some signal, but some delineation in the MIBG (Figure 2c); whilst the tumour is most clearly visible in the ^68^Ga Dotatate scan (Figure 2b). Plasma metanephrine levels showed (Figure 2f) rising normetanephrine and 3-methoxytramine levels over time with disease progression.

*T*_2_ weighted images (Figure 1), performed prior to the HP-MR acquisition, showed a large right-sided tumour near the adrenal gland (highlighted in green). The axial plane shows the tumour’s heterogeneous nature via a cystic portion, reflected by the brighter signal within the ROI.

**Figure 3.**
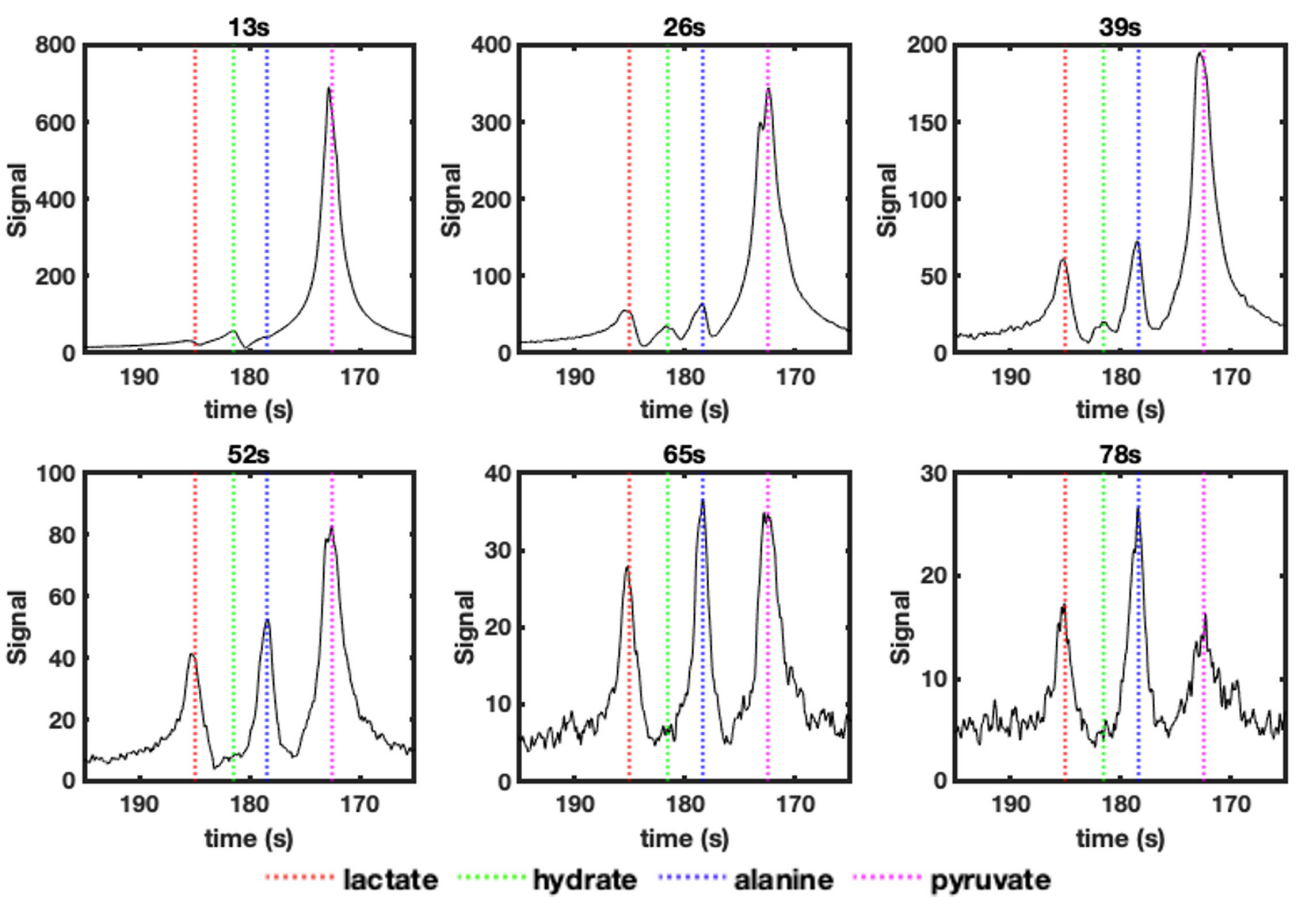
Change in nonlocalised spectroscopy signals, up to 78s after the completion of injection of hyperpolarised [1-^13^C] pyruvate. In each spectrum the peak positions for: [1-^13^C] lactate (185ppm – red dashed line), [1-^13^C] (pyruvate-)hydrate (181ppm – green dashed line), [1-^13^C] alanine (178ppm – blue dashed line) and [1-^13^C] pyruvate (172ppm – magenta dashed line) are shown.

Non-localised spectra (Figure 3) were acquired every 13 s to confirm the presence of hyperpolarised [1-^13^C] pyruvate (172 ppm) and its metabolites (Figure 3b); these were seen to be [1-^13^C] lactate: 185 ppm; [1-^13^C] pyruvate hydrate, also known as, 2,2-dihydroxypropanoic acid: 181 ppm; [1-^13^C] alanine: 178 ppm. [1-^13^C] Pyruvate hydrate disappears after 13 s, whilst [1-^13^C] lactate and [1-^13^C] alanine signals are visible for up to 65 s after the completion of injection. These chemical shifts were also used to resolve the bSSFP echo data to produce metabolite specific maps.

**Figure 4.**
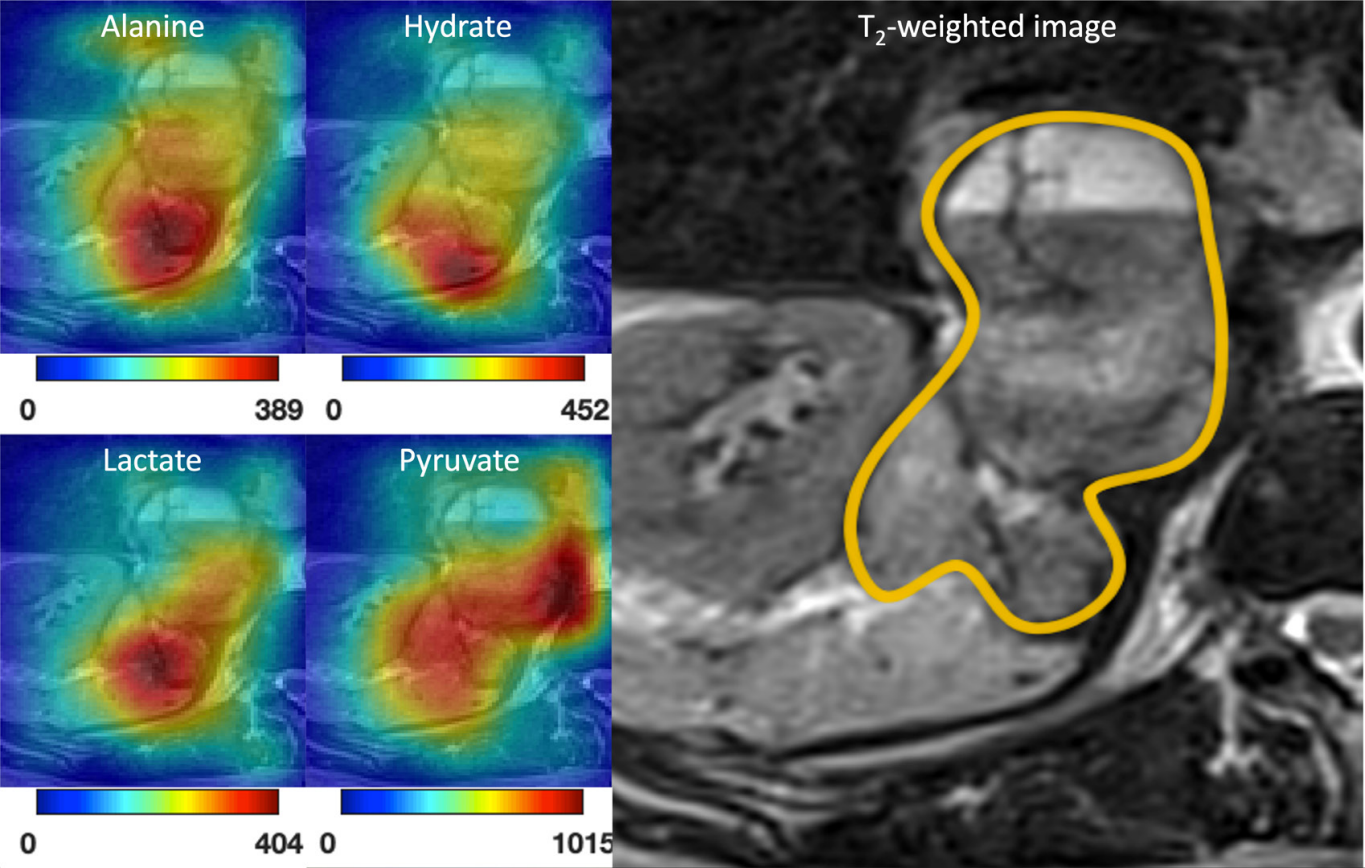
Total carbon signal maps for [1-^13^C] alanine, [1-^13^C] pyruvate hydrate, [1-^13^C] lactate and [1-^13^C] pyruvate, across the entire acquisition time frame, were overlaid on a T­-weighted TSE image (right). The PPGL’s location is outlined in orange.

**Figure 5.**
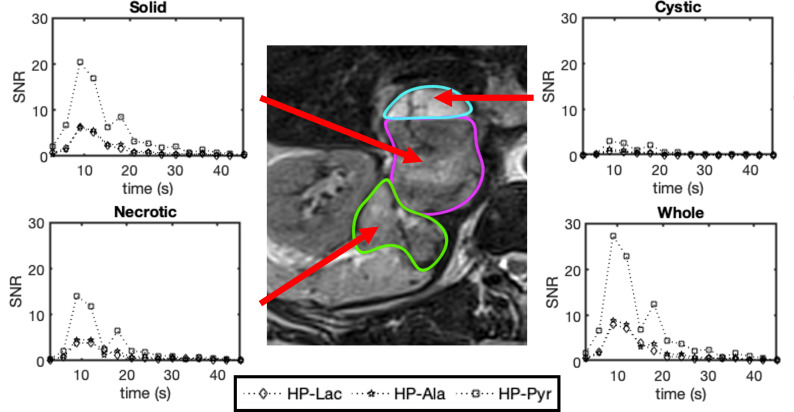
Change in metabolite signal to noise ratios for: [1-^13^C] lactate (HP-Lac), [1-^13^C] alanine (HP-Ala) and [1-^13^C] pyruvate (HP-Pyr) over time at the tumour site. The SNRs reflect the mean value from each ROI at each time point. The ‘Whole’ time point is a combination of the other three ROIs with the mean SNRs calculated at each time point.

Maps for total [1-^13^C] lactate, [1-^13^C] hydrate, [1-^13^C] alanine and [1-^13^C] pyruvate signals, obtained from the sum of signals over time, are shown overlaid on a *T*_2_ weighted image (Figure 4) of the pheochromocytoma. The pyruvate is shown to localise, primarily in the solid and necrotic portions of the tumour, with little signal in the cystic area (outlined in turquoise in Figure 5). Lactate, hydrate and alanine signals were seen to be strongly present in the necrotic region of the tumour (outline in green in Figure 5), with the solid tumour (marked out in magenta in Figure 5) also containing lactate and alanine. Low signal for all metabolites is seen in the cystic portion of the tumour, ROI analysis (Figure 5) reflects this with low metabolite SNRs seen in this ROI compared to the rest of the tumour.

The similar area under the curve (AUC) ratios across all ROIs suggest similar levels of activity for alanine aminotransferase (ALT) and lactate dehydrogenase (LDH) (Table 1). The necrotic tissue possesses a higher lactate to pyruvate and alanine to pyruvate AUC ratio than the solid region, whilst the cystic portion had the lowest values.

**Table 1.** Area under the curve ratios for lactate to pyruvate (Lac/Pyr AUC - middle column) and alanine to pyruvate (Ala/Pyr AUC - right hand column) signal to noise ratios (SNR)s, within the different ROIs identified (left hand column).

ROI	Lactate to pyruvate AUC	Alanine to pyruvate AUC
Whole tumor	0.33	0.36
Cystic	0.16	0.14
Solid	0.30	0.33
Necrotic	0.37	0.44

AUC, area under the curve; ROI, region of interest.

## Discussion

This study demonstrates the first in-human analysis of PPGL metabolism via HP-MR, with both non-localised spectroscopy and spectroscopic imaging data obtained. HP-MR provides an opportunity to dynamically assess metabolic processes and thereby enzymatic function. We observed, in real time, the conversion of hyperpolarized [1-^13^C] pyruvate to [1-^13^C] lactate, and from [1-^13^C] pyruvate to [1-^13^C] alanine, which we assume to be representative of LDH and ALT enzymatic activity, respectively.

LDH is the enzyme that interconverts pyruvate and lactate, and it also plays a role in regulating nutrient exchange between tumour and stroma. Higher levels of LDH are commonly found in tumours,^3,4^ and have been reported in PPGLs.^6^ It is therefore unsurprising that we observed [1-^13^C] lactate signal within tumour tissue (Figure 4).

An abundant supply of amino acids is critical for cancers to proliferate. A recent study demonstrated a correlation between cell proliferation and alanine generation in breast cancer cells, and although no evidence has been found to date for PPGLs, this could be a potential mechanistic explanation behind the presence of alanine we observed within our case.^7^

Under certain assumptions, the AUC ratio can be used as a simple model-free surrogate for enzyme activity.^8^ The similar lactate to pyruvate and alanine to pyruvate AUC ratios over the whole tumour (0.33 and 0.36 respectively) suggest that both LDH and ALT are important in PPGL metabolism. Such information on enzymatic activity is not available through PET tracers, which mainly concentrate on the relative uptake and accumulation of radioactivity within cells as opposed to real time metabolism.^1^ Moreover, with the advent of metabolomic analysis of tumours it may also be possible, in future trials, to correlate metabolic profiles with metabolic activity quantified by HP-MR.

A disadvantage of PET tracers is that uptake varies, depending on tumour type, across all radiotracers, as shown by the low-moderate avidity for ^18^F-FDG and MIBG (Figure 2c-d). This may require a subject being exposed to multiple ionising tracers to delineate their tumours. HP-MR, as shown here, does not discriminate against tumour type, allowing for a single scan to be used to assess the metabolism of different tumour types.^4,9,10^

Notably, this study was performed after the subject had undergone three cycles of TMZ treatment, which may have influenced tumour metabolism. Without a baseline scan before therapy, we cannot speculate with respect to the influence the treatment may have had on the scans presented. As this was primarily a proof-of-concept study any further trials should involve hyperpolarised ^13^C-MR scans before and after therapy.

Limitations of this study include the fact that the bSSFP sequence used involved the acquisition of only a single slice, limiting the spatial information obtained. With such a large tumour (12 cm), a volumetric imaging sequence would provide an interesting opportunity to visualise the heterogeneity, if any, across individual ROIs. Similarly, the spatial resolution of these acquisitions, in comparison to clinically used sequences, was very coarse at 21 × 21 x 30 mm per voxel, and an improvement of this would allow for better localisation of the metabolite signals to different tissue types.

Further studies will be necessary to define the role of HP-MR in PPGL-associated disease with respect to diagnostics, prognostication, and longer-term disease surveillance. This will include defining the significance of the metabolite signals and the modality’s ability to differentiate PPGLs according to size, anatomical position, metastatic potential, and response to therapy. HP-MR may provide a prognostic tool to risk stratify for more aggressive PPGLs as well as identifying which patients require closer, longer-term follow-up.

## Learning points

HP-MR may provide an opportunity to observe real-time PPGL metabolism.
