# Supplementary material for: First-in-human in-vivo depiction of paraganglioma metabolism by hyperpolarised 13C-magnetic resonance
Source: BJR Case Rep. 2023 Sep 28;9(6):20220089. doi: 10.1259/bjrcr.20220089 (PMC10621573; doi:10.1259/bjrcr.20220089)
Supplement: Supplementary Material 1. [file bjrcr.20220089.suppl-01.docx]

**Supplementary information**

**Pulse sequence parameters**

**^1^H-imaging**

The tumour position was anatomically localised using T_2_-weighted scans, performed in the axial: field of view: 250x250x30mm; matrix size: 192x192x10; slice thickness: 30mm; TR: 5640ms; ΔTE: 108ms; No. of echoes: 15; flip angle: 90°; NSA: 3 and coronal: field of view: 460x370x30mm; matrix size: 256x206x10; slice thickness: 30mm; TR: 5630ms; ΔTE: 107ms; No. of echoes: 15; flip angle: 90°; NSA: 3; planes.

Prior to contrast injection a dual echo gradient sequence: field of view: 250x250x30mm; matrix size: 256x256x1; slice thickness: 30mm; TR: 4.8ms; ΔTE: 107ms; No. of echoes: 2; flip angle: 15°; NSA: 1; was employed to assess the magnetic field inhomogeneity across the field of view. A field map was calculated from this and used in post-processing as part of a custom IDEAL algorithm.

**^13^C-imaging**

Four consecutive 2D multiecho-balanced steady state free precession (me-bSSFP) acquisitions with: field of view: 250x250x30mm; matrix size: 12x12x10; slice thickness: 30mm; TR: 15.8ms; ΔTE: 11ms; No. of echoes: 7; flip angle: 30°; NSA: 16; were acquired immediately after the completion of injection.

A nonlocalized spectroscopy sequence: TR: 1ms; flip angle: 10°; NSA: 1, BW: 4000Hz was then acquired. This was followed by another set of four me-bSSFP acquisitions and an FID. This was repeated for a total acquisition time of 2m30s.
